# The Extended Family of CD1d-Restricted NKT Cells: Sifting through a Mixed Bag of TCRs, Antigens, and Functions

**DOI:** 10.3389/fimmu.2015.00362

**Published:** 2015-07-28

**Authors:** Elodie Macho-Fernandez, Manfred Brigl

**Affiliations:** ^1^Department of Pathology, Brigham and Women’s Hospital, Harvard Medical School, Boston, MA, USA

**Keywords:** CD1d, antigen presentation, lipid antigens, glycosphingolipids, phospholipids, inflammation, infection, cancer

## Abstract

Natural killer T (NKT) cells comprise a family of specialized T cells that recognize lipid antigens presented by CD1d. Based on their T cell receptor (TCR) usage and antigen specificities, CD1d-restricted NKT cells have been divided into two main subsets: type I NKT cells that use a canonical invariant TCR α-chain and recognize α-galactosylceramide (α-GalCer), and type II NKT cells that use a more diverse αβ TCR repertoire and do not recognize α-GalCer. In addition, α-GalCer-reactive NKT cells that use non-canonical αβ TCRs and CD1d-restricted T cells that use γδ or δ/αβ TCRs have recently been identified, revealing further diversity among CD1d-restricted T cells. Importantly, in addition to their distinct antigen specificities, functional differences are beginning to emerge between the different members of the CD1d-restricted T cell family. In this review, while using type I NKT cells as comparison, we will focus on type II NKT cells and the other non-invariant CD1d-restricted T cell subsets, and discuss our current understanding of the antigens they recognize, the formation of stimulatory CD1d/antigen complexes, the modes of TCR-mediated antigen recognition, and the mechanisms and consequences of their activation that underlie their function in antimicrobial responses, anti-tumor immunity, and autoimmunity.

## Introduction

Traditionally, the immune system has been separated into innate and adaptive immunity. However, in an unconventional way, innate T cells such as CD1d-restricted natural killer T (NKT) cells, MR1-restricted mucosal-associated invariant T (MAIT) cells, γδ T cells, and some CD1a, b, c-restricted T cells share features of both innate and adaptive immune cells, allowing them to form a critical bridge between the two arms of the immune system (1–4). NKT cells recognize lipid antigens presented by the monomorphic MHC class-I-like molecule CD1d and are highly conserved in humans and mice. In response to a wide range of self- and foreign antigens, NKT cells are activated rapidly and exhibit both pro-inflammatory and immunoregulatory characteristics, resulting in either protective or harmful roles in numerous pathological states in mice and humans, including microbial infection, autoimmune disease, allergic disease, and cancer (5–10). Therefore, NKT cells represent an immunotherapeutic target with broad clinical potential. CD1d-restricted T cells can be divided into four main subsets based on their T cell receptor (TCR) usage and antigen specificities (Table 1). Type I (or invariant) NKT cells constitute the first and best characterized subset, and use an invariant TCRα chain (Vα14-Jα18 in mice and Vα24-Jα18 in humans). In addition, there are CD1d-restricted T cells that use a more varied TCR repertoire, including Vα24^−^ and Vα10^+^ NKT cells in mice and humans, respectively, diverse or type II NKT cells, as well as T cells expressing γδ or δ/αβ TCRs. In this review, while using type I NKT cells as comparison, we will focus on type II NKT cells and the other non-invariant CD1d-restricted T cell populations and discuss our emerging understanding of their TCR usage, antigen specificities, mode of antigen/CD1d recognition, innate-like mechanisms of activation, and their immunological functions.

**Table 1:** **Classification of CD1d-restricted T cells**.

	Type I NKT cells	Vα24^−^ (h) and Vα10^+^ (m) NKT cells	Type II NKT cells	γδ and δ/αβ T cells
TCR repertoire	m: invariant (i)Vα14Jα18, Vβ8, Vβ7 or Vβ2	m: iVα10Jα50, Vβ8	m: diverse with oligoclonal Vα3.2-Jα9/Vβ8 and Vα8/Vβ8	m: Vδ4^+^
	h: iVα24Jα18, Vβ11	h: limited α chains (Vα10, Vα2 or Vα3), Vβ11	h: diverse?	h: Vδ1^+^, Vδ3^+^

α-GalCer reactivity	all	all	no	some

Sulfatide reactivity	no	no	some	some

Other antigens	Microbial α-GSL, α-GDAG	Microbial α-GSL	Mammalian and microbial phospholipids (PG, DPG, PI)	Mammalian and pollen phospholipids (PE, PC, DPG)
	Mammalian β-GSL, phospholipids, lysophospholipids, plasmalogens	Mammalian β-GSL and β-GlcSph	Mammalian lysophospholipids (LPC, LSM, LPE, β-GlcSph)	

Function	NKT1 (IFN-γ, IL-2)	T_H_1-like (IFN-γ)	T_H_1-like (IFN-γ, IL-2)	T_H_1-like (IFN-γ)
	NKT2 (IL-4, IL-9, IL-10, IL-13)	T_H_2-like (IL-4, IL-13)	T_H_2-like (IL-4, IL-10, IL-13)	T_H_2-like (IL-4)
	NKT17 (IL-17A, IL-21, IL-22)	T_H_17-like (IL-17)	T_H_17-like (IL-17)	
	NKT_FH_-like (IL-5, IL-6, IL-10, IL-17)		Cytotoxicity (perforin, granzyme)	
	Cytotoxicity (perforin, granzyme)		T_FH_-like (IL-5, IL-6, IL-10, IL-17)	

Phenotype	Activated/memory	Activated/memory some naïve?	Activated/memory some naïve?	Activated/memory

*α-GalCer, α-galacotsylceramide; α-GSL, α-glycosphingolipid; α-GDAG, α-glyco(Gal/Glc)diacylglycerol; β-GlcSph,β-glucosylsphingosine; DPG, diphosphatidylglycerol; h, human; LPC, lysophosphatidylcholine; LPE, lysophosphatidylethanolamine; LSM, lysosphingomyelin; m, mouse; PG, phosphatidylglycerol; NKT, natural killer T cell; TCR, T cell receptor*.

## The CD1d-Restricted T Cell Family

### Type I NKT cells

The discovery of the first CD1d-presented antigen, α-galactosylceramide (α-GalCer), by Kawano and colleagues in 1997 enabled several important steps forward in our understanding of NKT cell biology, particularly of type I or invariant NKT (iNKT) cells (11). Type I NKT cells express an invariant Vα14Jα18 TCR α-chain in mice and Vα24Jα18 in humans, paired with a limited repertoire of TCR β-chains (Vβ8, Vβ7, Vβ2 in mice and exclusively Vβ11 in humans) (Table 1). Type I NKT cells are highly autoreactive even at steady state and display an activated/memory phenotype with high surface levels of the activation markers CD69, CD44, and CD122 (IL-2R β-chain) and low expression of CD62L, a marker expressed by naïve T cells that home to lymph nodes (12, 13). The use of mice that are deficient in CD1d (lack type I and type II NKT cells) or selectively deficient in type I NKT cells (Jα18^−/−^), administration of α-GalCer to activate type I NKT cells *in vivo*, and the ability to track type I NKT cells with fluorescent CD1d/α-GalCer tetramers has allowed the elucidation of many functions of type I NKT cells *in vivo*. Type I NKT cells play critical roles in local and systemic immune responses and are essential for controlling tumor development and antimicrobial immune responses. They can also exert detrimental effects in the pathogenesis of autoimmune and allergic disorders. The biology of type I NKT cells has been extensively covered in several excellent recent reviews (2, 3, 14–16), including their roles in microbial infections (2, 7, 17–20), autoimmunity and inflammation (9, 21, 22), and tumor immunity (23). This broad range of type I NKT cell functions relies on their rapid secretion of copious amounts of various cytokines, including IFN-γ, IL-2, IL-4, IL-9, IL-10, IL-13, IL-17, IL-21, and GM-CSF (24–26), and their interactions with other immune cells (2, 16). Based on the differential expression of cytokines, transcription factors and surface markers, several functionally distinct type I NKT cell subsets have been described in humans and mice (Table 1). Human type I NKT cells expressing CD4 produce Th2-type cytokines whereas both CD4^+^ and CD4^−^ subsets can generate Th1-type cytokines and secrete cytotoxic molecules such as perforin and granzyme (24, 27). In C57BL/6 mice, Th1-like type I NKT cells (also referred to as NKT1 cells) represent the majority of type I NKT cells in liver and spleen, are characterized by the Th1-associated transcription factor T-bet, mostly express NK1.1 and IL-12 receptor (IL-12R) and their pronounced production of IFN-γ is critical for their function during various immune responses, including anti-tumor immunity (24, 28, 29). Th2-like type I NKT cells (NKT2 cells) are the most abundant type I NKT cell subset in BALB/c mice and are enriched in lung and intestine of C57BL/6 mice. NKT2 cells have been reported to play an important role in Th2-mediated diseases through the secretion of IL-4, IL-9, IL-10, and IL-13 (30, 31). Th17-like type I NKT cells (NKT17 cells) are primarily found in lung, skin and peripheral lymph nodes, produce IL-17A and IL-22 (26, 32, 33), and express the retinoic acid receptor-related orphan receptor γt (RORγt) transcription factor (34). Finally, a small number of type I NKT cells with developmental, phenotypical, and functional features of follicular helper (35) T cells secrete IL-21, support the formation of germinal centers (36–38) and require the transcription factor Bcl-6 for their development (36). Despite their varied functional differentiation, most type I NKT cell subsets express the transcription factors PLZF, which is fundamental for their development, and GATA3 (39). However, instead of PLZF, a distinct type I NKT cell population, newly referred to as NKT10 cells, express the transcription factor E4BP4 which confers their regulatory properties (40, 41). Thus, the remarkable functional versatility of type I NKT cells during various immune responses can at least in part be explained by the existence of functionally distinct subsets, while organ-specific functions and plasticity of type I NKT cell subsets have not been adequately investigated.

### α-GalCer-reactive Vα24^−^ and Vα10^+^ NKT cells

Recognition of α-GalCer by type I NKT cells was thought to be highly correlated with expression of the invariant Vα14-Jα18 or Vα24-Jα18 TCR α-chains in mice and humans, respectively. However, α-GalCer-reactive, CD1d-restricted NKT cells that use different TCR α-chains have subsequently been described in both mice and humans. In humans, one study using α-GalCer-loaded CD1d tetramers found Vα24-negative T cell populations expressing Vα10, Vα2, or Vα3 joined to Jα18 and paired with Vβ11 (42) whereas another study found a diversity of Vα24^−^ Jα18^−^/Vβ11^−^ TCRs that stained with α-GalCer/CD1d tetramers (Table 1) (42–44). Despite their equivalent reactivity to α-GalCer or bacterial α-linked glycosphingolipids (GSLs), Vα24-negative subset displayed preferential antigen specificities for α-GlcCer (42). Surprisingly, a Vα24^−^/CD1d-α-GalCer^+^ population was found to predominantly display a naïve phenotype and low or intermediate expression level of PLZF (45). So far, data regarding the function of these NKT cells in humans are limited.

Similarly, in mice, CD1d-restricted NKT cells that recognize α-GalCer but do not use the canonical Vα14-Jα18 TCR α-chain have also been described, and predominantly express a semi-invariant Vα10-Jα50 TCR α-chain paired with a Vβ8^+^ TCR β-chain (Vβ8.1/0.2 or Vβ8.3). Vα10^+^ NKT cells have been identified in thymus, spleen, and liver of Jα18^−/−^ mice by CD1d-α-GalCer tetramer staining, and displayed a CD44^high^ CD69^int^ pre-activated phenotype, similar to type I NKT cells. Like other NKT cell populations, their development required expression of CD1d. In addition to α-GalCer, Vα10^+^ NKT cells preferentially recognize other glucose-based glycolipids such as α-GlcCer, GSL-1 from *Sphingomonas* or α-GlcA-DAG from *Mycobacterium smegmatis*, and in response to stimulation with these glycolipids produce large amounts of cytokines including IFN-γ, IL-4, IL-13, and IL-17 (46). Thus, recognition of CD1d/α-GalCer complexes by NKT cells in humans and mice is not uniformly restricted to the use of Vα24-Jα18/Vβ11 or Vα14-Jα18 TCRs, respectively, but can be mediated by a range of Vα and Vβ domains, highlighting the variation in antigen recognition among CD1d-restricted α-GalCer-reactive TCRs. Furthermore, the different antigen specificities of some of these non-canonical α-GalCer-reactive NKT cells may correlate with distinct functional capabilities, and it remains to be explored if this subset harbors NKT cells that can be expanded *in vivo* and form memory responses.

### Type II NKT cells

CD1d-restricted T cells that do not express the Vα14-Jα18 rearrangement and do not recognize α-GalCer were first described in MHC II-deficient mice among the remaining CD4^+^ T cells (47). From then called diverse NKT (dNKT), type II NKT, or variant NKT (vNKT) cells, this NKT cell population, found in both mice and humans, exhibits a more heterogeneous TCR repertoire (Table 1). For example in mice, the type II NKT cells that have been described use Vα1, Vα3, Vα8, or Vα11 TCR α-chains paired with Vβ8 or Vβ3 TCR β-chains, or Vα4 paired with Vβ5 or Vβ11, and appear to contain oligoclonal Vα3.2-Jα9/Vβ8 and Vα8/Vβ8 TCR families (48–50). Currently, no direct and specific tools exist to identify the entire type II NKT cell population *in vivo*, but different approaches have been developed to study these cells in mice. The first is to compare the immune responses between Jα18^−/−^ mice (lacking only type I NKT cells) and CD1d^−/−^ mice (lacking both type I and type II NKT cells). It should be noted that Jα18^−/−^ mice exhibit lower TCR repertoire diversity due to deficits in rearrangements of several Jα segments (51). This raises the possibility that defects described in Jα18^−/−^ mice are not solely due to type I NKT cell deficiency and a more specific type I NKT cell-deficient mouse model is needed. The second tool used to study type II NKT cell function is 24αβ TCR transgenic mice that were generated by overexpressing the Vα3.2/Vβ9 TCR from the type II NKT cell hybridoma VIII24 (52). A third approach is the use of Jα18-deficient IL-4 GFP (Jα18^−/−^ 4get) reporter mice. This model is based on the finding that some type II NKT cells spontaneously express IL-4 mRNA transcripts, similar to type I NKT cells (53, 54). However, this approach does not identify all type II NKT cells since, for example, GFP^+^ type II NKT cells from Jα18^−/−^ 4get mice do not respond to sulfatide, an antigen that is recognized by a significant number of type II NKT cells. The fourth approach to identify type II NKT cells is the use of lipid antigen-loaded CD1d tetramer reagents, similar to the use of α-GalCer/CD1d tetramers that are used to detect type I NKT cells. The discovery of sulfatide as a potent type II NKT cell ligand led to the generation of sulfatide-loaded CD1d tetramers and revealed an oligoclonal TCR repertoire among sulfatide-specific type II NKT cells with predominant use of Vα3/Vα1-Jα7/Jα9 and Vβ8.1/Vβ3.1-Jβ2.7 genes (50, 55, 56). However, sulfatide/CD1d tetramers appear to be more difficult to use compared to α-GalCer/CD1d tetramers, likely due to difficulties in loading the antigen and/or greater instability of sulfatide/CD1d complexes and, moreover, not all type II NKT cells recognize sulfatide (50, 54). Similar to sulfatide, β-glucosylceramide (β-GlcCer)- or glucosylsphingosine β-GlcSph-loaded CD1d tetramers stained a subset of human and mouse type II NKT cells (57). Furthermore, the recent discovery of several microbial antigens recognized by different type II NKT hybridomas enables the design of antigen-loaded CD1d tetramers that are likely to be useful to characterize type II NKT cells *in vivo* (58, 59). Another approach to study type II NKT cells is the use of dNKT hybridomas that were initially identified by their recognition of CD1d-expressing APC and their use of TCR α-chains different from Vα14-Jα18 (47–49, 60, 61). These dNKT hybridomas were used to characterize the TCRs expressed by type II NKT cells and continue to be used to identify self- and microbial lipid antigens that are recognized by type II NKT cells.

Using the approaches described above, many type II dNKT cells appear to share phenotypic and functional features with type I NKT cells such as high autoreactivity (62), PLZF- and SAP-dependent thymic development (54, 63), constitutive expression of IL-4 mRNA (54), and the ability to secrete a wide range of cytokines rapidly after stimulation, including IFN-γ, IL-2, IL-4, IL-10, IL-17, GM-CSF, and cytolytic mediators such as perforin (54, 63). Furthermore, many type II NKT cells have a CD44^high^ CD69^+^ CD122^+^ activated/memory phenotype, whereas CD62L is more or less expressed dependent on which transgenic mouse model is used, and can be divided into different subsets depending on CD4 and NK1.1 expressions (54, 63–65). However, several studies suggest that type II NKT cells exist that are phenotypically and functionally distinct from type I NKT cells. For example, most of the T cells stained with sulfatide/CD1d tetramers in C57BL/6 mice did not express the early activation marker CD69 (50). Moreover, in 24αβ TCR transgenic mice on the non-obese diabetic (NOD) background, the majority of DN 24αβ NKT cells were CD44^int^, CD45RB^high^, CD62L^high^, CD69^−/low^, similar to conventional T cells, whereas the majority of CD4^+^ 24αβ NKT cells exhibited the typical type I NKT CD44^high^, CD45RB^low^, CD62L^low^, CD69^high^ activated/memory phenotype (66). In addition, in both humans and mice, type II NKT-T_FH_ populations have recently been described that recognized β-GlcCer or β-GlcSph (57). The human type II NKT-T_FH_ population utilized Vα24^−^/Vβ11^−^ TCRs with diverse Vβ chains and displayed a naïve CD45RA^+^, CD45RO^−^, CD62^high^, CD69^−/low^ phenotype. The majority of these cells expressed a T_FH_-like phenotype in mice and humans (CXCR5^+^, PD-1^high^, ICOS^high^, Bcl6^high^, FoxP3^−^, IL-21^+^) at steady state and mainly secreted IL-5, IL-6, IL-10, and IL-17 following activation. Their T_FH_ properties were associated with the induction of GC B cells and lipid-specific antibodies *in vivo* in a CD1d-dependent manner.

In humans, CD1d-restricted type II NKT cells appear to be much more frequent than type I NKT cells. In human bone marrow, approximately 25% of CD3^+^ T cells expressed CD161 and half of the CD161^+^CD3^+^ cells recognized CD1d. Interestingly, the majority of these CD1d-restricted T cells used Vα24^−^/Vβ11^−^ TCRs (67). In PBMC of healthy individuals, approximately 0.5% of CD3^+^ lymphocytes stained with β-GlcCer/CD1d tetramers, similar to numbers in Gaucher’s disease patients, whereas 1–2% of CD3^+^ lymphocytes in these patients stained positive with β-GlcSph/CD1d tetramers, compared to 0.2% in healthy individuals (57). In myeloma patients, lysophosphatidylcholine (LPC)-loaded CD1d dimers stained on average 0.6% of T cells in PBMC, several fold higher than type I NKT cell numbers determined with α-GalCer-loaded CD1d dimers (68), whereas in healthy controls, both LPC- and α-GalCer-loaded CD1d dimers stained approximately 0.05% of PBMC.

Thus, type II NKT cells can be distinguished from type I NKT cells by their use of more diverse TCRs and their distinct antigen specificities. Many type II NKT cells are phenotypically and functionally similar to type I NKT cells, however, some type II NKT cells appear to have a naïve T cell phenotype and further studies are required to test if these cells are capable to form antigen-specific memory responses. In addition, similar to type I NKT cells, type II NKT cells may also harbor functionally distinct subsets, and it will be important to determine to what extent functional differentiation correlates with antigen specificities.

### CD1d-restricted γδ and δ/αβ T cells

In addition to the use of αβ TCRs, CD1d-restricted T cells expressing γδ TCRs have recently been described in both mice and humans (Table 1). According to their Vδ-chain expression, human γδ T cells can be divided into two major populations: Vδ2^+^ and “non-Vδ2” subsets, the latter comprise Vδ1^+^ γδ T cells and the less prevalent Vδ3^+^ γδ T cells (69, 70). Vδ1^+^ γδ T cells are mainly tissue-resident and are found in the skin and at mucosal surfaces, whereas Vδ2^+^ γδ T cells are predominant in human blood. Compared with αβ T cells, the types of antigens recognized by γδ T cells and the role and function of antigen presentation in γδ TCR recognition are much less clear. Interestingly, some γδ T cells have recently been found to directly recognize CD1d-presented lipid antigens (71). Indeed, Spinazzo and colleagues showed that only γδ T cells, but not αβ T cells, from peripheral blood and nasal mucosa of cypress pollen-sensitive subjects were activated in a CD1d-dependent manner by phospholipids extracted from pollen grains (72–74). Up to now thought of as type I NKT cell-specific ligands, α-GalCer and some of its derivatives including OCH were recognized by a subset of circulating human Vδ1^+^ γδ T cells (75). Similarly, sulfatide-reactive CD1d-restricted Vδ1^+^ γδ T cells were found in human blood and among gut T cells (76, 77). In addition, Vδ3^+^ γδ T cells found in the liver of patients with leukemia or chronic viral infection recognized CD1d molecules and killed CD1d^+^ cells (78). In mice, development of myocarditis following infection with coxsackievirus B3 (CVB3) relied on CD1d up-regulation and CD1d-dependent activation of Vγ4^+^ γδ T cells (79). Furthermore, α-GalCer reactive, CD1d-restricted T cells using TCRs in which the Vδ1 gene is fused to Jα and Cα domains that are paired with an array of TCR β-chains to form a δ/αβ TCR have recently been described (80). Thus, lipid-specific γδ and δ/αβ TCRs expand the TCR diversity among CD1d-restricted T cells. Understanding the full range of antigens that are recognized by CD1d-restricted T cells that use TCR γ/δ genes, the extent of permissive TCR diversity and the numbers of these cells under physiologic conditions and during infection and other pathologies constitute important areas for future exploration.

## Mechanisms of CD1d-Restricted T Cell Activation

In contrast to the high degree of TCR diversity and antigen specificity of adaptive MHC-restricted T cells, CD1d-restricted T cells utilize a more restricted TCR repertoire and recognize antigens presented by a monomorphic antigen-presenting molecule. Yet, these cells are able to respond to highly diverse infectious agents and become activated in a variety of non-infectious pathological conditions. Recent progress has started to shed light on how the self- and exogenous lipid antigens that are presented by CD1d, the molecular underpinnings of TCR-mediated recognition of lipid/CD1d complexes, the antigen-presenting pathways that result in formation of stimulatory lipid/CD1d complexes, and the non-TCR signals all contribute to the activation of type II NKT cells and other non-invariant CD1d-restricted T cell populations.

### CD1d-presented ligands

**Figure 1:**
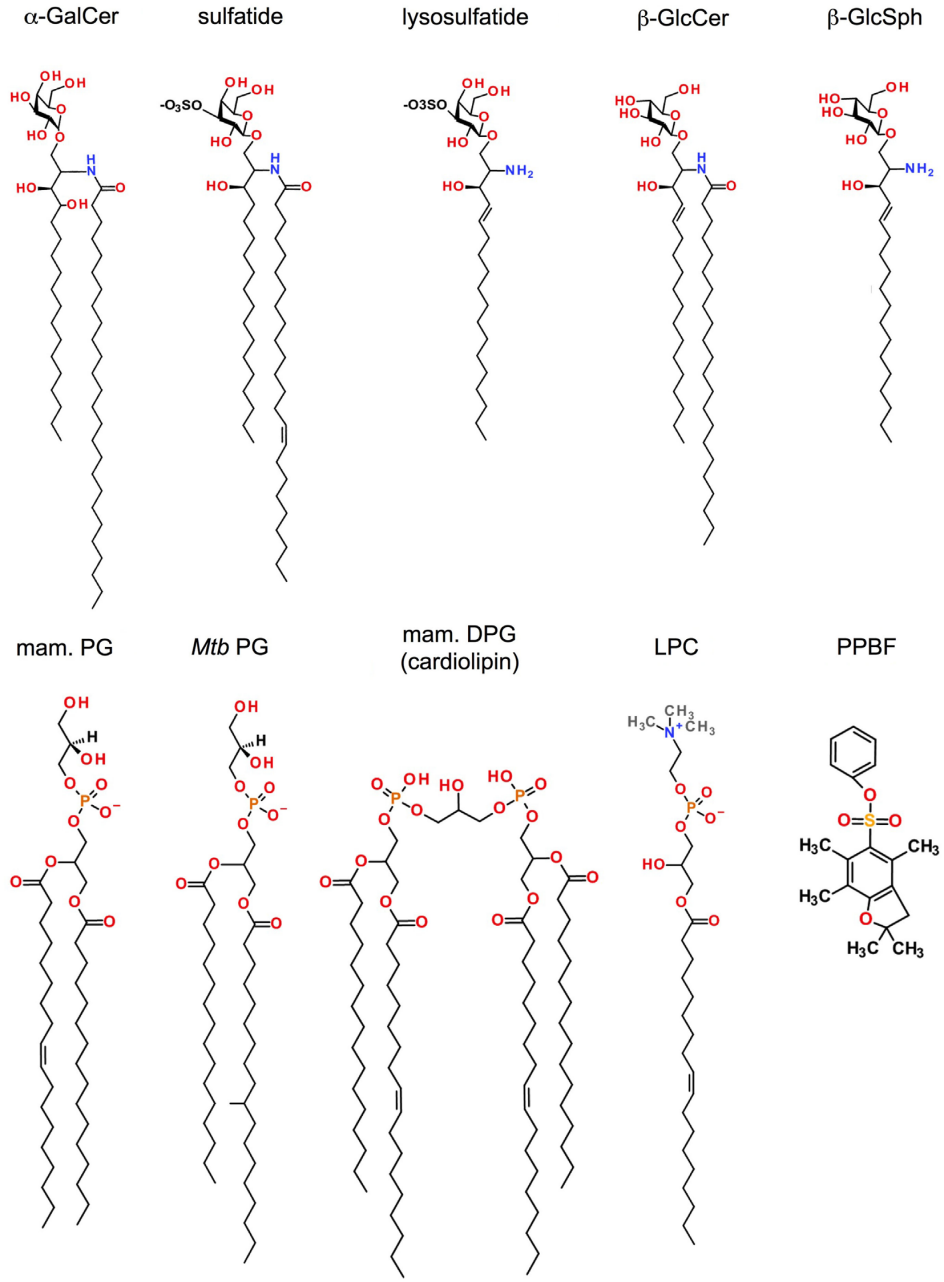
**Antigens recognized by CD1d-restricted T cells**. Shown are the structures of lipid antigens for type I and/or type II NKT cells: the prototypical type I NKT cell glycolipid α-galactosylceramide (α-GalCer); the lipid self-antigen sulfatide (type II NKT cells); the mammalian phosphatidylglycerol (PG; type I and type II NKT cells) and its microbial counterparts from *Corynebacterium glutamicum* (*Cg*) or *Mycobacterium tuberculosis* (*Mtb*) (type II NKT cells); the mammalian diphosphatidylglycerol (DPG or cardiolipin) whose *Mtb* counterpart shares the same alkyl chains as *Mtb* PG (Type II NKT cells); lysophosphotidylethanolamine (lysoPE; type I and type II NKT cells), lysophasphatidylcholine (LysoPC; type I and type II NKT cells), and phenyl 2,2,4,6,7-pentamethyldihydrobenzofuran-5-sulfonate (PPBF, type II NKT cells).

**Figure 2:**
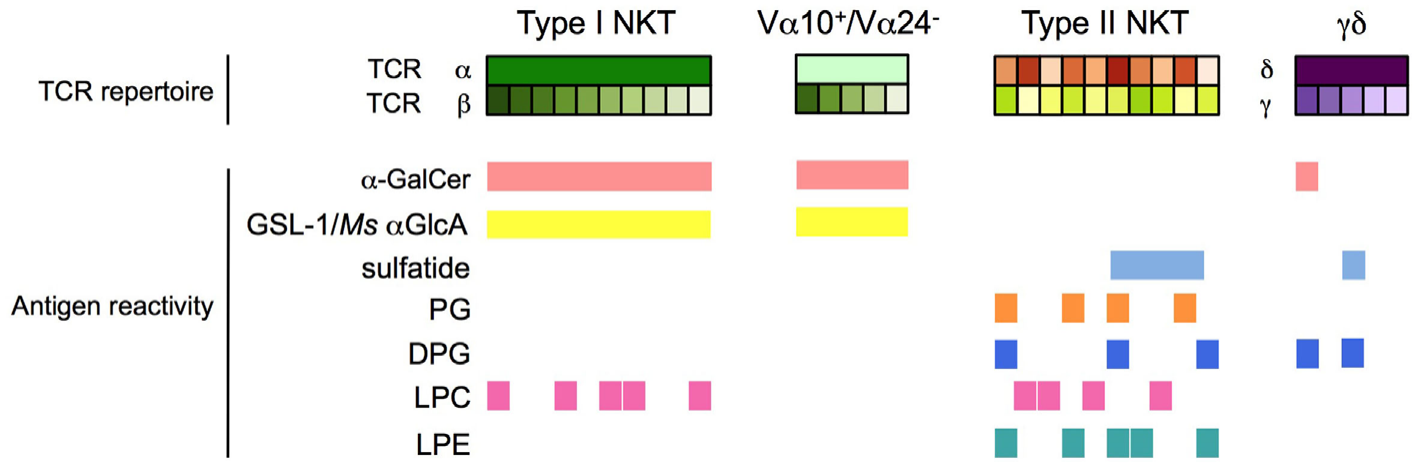
**Antigen specificity of CD1d-restricted T cells**. Type I NKT cells, Vα24^−^ (human) and Vα10^+^ (mouse) NKT cells, type II NKT cells, and CD1d-restricted γδ and δ/αβ T cells have distinct and partially overlapping antigen specificities. h, human; m: mouse; *Ms*, *Mycobacterium smegmatis*.

α-galactosylceramide and numerous related derivatives have helped to define the range of antigen specificity among type I NKT cells and provide powerful agents for their pharmacologic stimulation with various functional outcomes (Figure 1). Furthermore, several α-linked GSLs and diacylglycerols that stimulate type I NKT cells have been isolated from various microbes, confirming that α-linked GSLs are indeed important natural antigens for type I NKT cells. In addition, an intensive search for the self-antigens that are critical for type I NKT cell development has revealed several candidates, including iGb3, β- and α-linked GSLs, and various phospholipids. All of these antigens have been the topic of excellent recent reviews and will not be discussed in detail here (81–84). Similarly, a number of endogenous and exogenous glycerol- or ceramide-based lipid antigens that stimulate non-invariant CD1d-restricted T cells have been identified, but significant differences in antigen specificity exist between the various CD1d-restricted T cell subsets as a result of their varied TCR usage. For example, type II NKT cells display specificities for antigens that are not typically considered to be agonists for type I NKT cells, but some overlap in antigen specificity between these two NKT cell subsets is becoming apparent. In contrast, only subtle differences in antigen specificity have been observed between type I NKT cells and the non-canonical Vα24^−^ NKT cells and Vα10^+^ in humans and mice, respectively. Interestingly, CD1d-restricted T cells with γ/δ TCRs have been identified that recognize α-GalCer or sulfatide, antigens that were thought to be specific for type I and type II NKT cells, respectively. Thus, antigen specificity alone is not a reliable criterion to define CD1d-restricted T cell subsets (Table 1; Figure 2).

#### Self-Ligands

Type II NKT cells were originally identified based on their autoreactivity to CD1d-expressing cells and subsequently individual stimulatory self-lipid antigens were identified. In addition, numerous studies have now demonstrated the activation of non-invariant CD1d-restricted T cells in diseases where foreign lipids are not present, such as autoimmunity, cancer, viral infection, or in response to toll-like receptor agonists, suggesting that the recognition of self-lipids is critical for their peripheral activation.

Sulfatide (SO_3_-3Galβ1Cer) is a self-GSL recognized by a subset of type II NKT cells that predominantly express Vα3/Vα1 and Vβ8.1/Vβ3.1 TCRs (Figure 1) (50, 55). Native sulfatide is a mixture of multiple sulfatide isoforms with different lengths and saturation of their fatty acid chains, and is found in many organs (brain, kidney, pancreatic β-cells) and tumor cells. A major component of the myelin sheets of the central nervous system, the mono-unsaturated C24:1 sulfatide isoform activates XV19 type II NKT cells in a CD1d-dependant manner, unlike its saturated (C24:0) or shortened (C18:1) isoforms. Although found in the nervous system in only minor quantities, the lysosulfatide (LSF) that lacks the entire fatty acid chain, is the most potent sulfatide isoform described (85). However, the XV19 type II NKT hybridoma exhibits high autoreactivity toward splenocytes from CST^−/−^ mice that lack cerebroside sulfotransferase and therefore cannot generate sulfatide, and sulfatide-reactive NKT cells were still detected in CST^−/−^ mice (55), indicating that sulfatide is not required for type II NKT cell development. Administration of sulfatide to mice has widely been used to study the function of sulfatide-specific type II NKT cells *in vivo*. In humans, the majority of T cells in PBMC that stained with sulfatide-loaded CD1d tetramers were γδ T cells that use a semi-invariant Vδ1^+^ γδ TCR (76).

Another β-linked GSL, β-glucosylceramide (β-GlcCer), has recently been shown to activate type I and type II NKT cells (Figure 1). Stimulation of APC by diverse TLR agonists can modify the GSL biosynthesis pathway by enhancing the expression of several glycosyltransferases and, as a consequence, the accumulation of endogenous GSL, including β-GlcCer, that are then presented by CD1d (86, 87). However, two recent studies have questioned the role of β-GlcCer in the activation of type I NKT cells. The studies attributed the stimulatory activity of synthetic or mammalian β-GlcCer to co-purified α-GlcCer and suggest that mammalian immune cells produce constitutively small quantities of stimulatory α-glycosylceramides under control of catabolic enzymes of the ceramide and glycolipid pathway (88, 89). For type II NKT cells, the β-GlcCer-22:0 isoform and its deacylated product, glucosylsphingosine or β-GlcSph, have been shown to activate type II NKT subsets in humans and mice and CD1d tetramers loaded with these antigens can be used to stain these cells (57). In another example of induced self-lipid-mediated activation, Zhao and co-workers showed that type II NKT cells in the Jα18-deficient IL-4 reporter mouse were activated in response to CpG ODN, a potent synthetic agonist of TLR9, which mimics a hallmark of microbial DNA (54). Similar to what has been observed for type I NKT cells (86), type II NKT cell activation by CpG ODN resulted in production of IFN-γ but not IL-4 or IL-13, and was partially CD1d-dependent. Type II NKT cells isolated from Jα18-deficient IL-4 reporter mice recognized β-GlcCer but not sulfatide or phospholipids. However, the lipids responsible for the activation of type II NKT cells following CpG ODN stimulation were not identified. Several type II NKT hybridomas (i.e., XV19, VIII24, VII57) were unresponsive to α-glycosylceramides such as α-GclCer (90) and autoreactivity of the XV19 type II NKT cell hybridoma persisted with use of β-GlcCer- or GSL-deficient cell lines, suggesting that glycolipids are not the only self-lipids responsible for the autoreactivity of type II NKT cells (91).

Mammalian phospholipids are a major component of cell membranes and have been identified as natural antigens that are recognized by CD1d-restricted NKT cells. Mouse CD1d-restricted type I and type II NKT cell hybridomas exhibit distinct but overlapping antigen recognition specificities, particularly in response to phosphatidylinositol (PI), phosphatidylethanolamine (PE), and phosphatidylglycerol (PG) (Figure 1) (62). Mammalian phospholipids differ from their microbial counterparts in the fatty acyl chain composition or the position of the fatty acyl molecules at the *sn*-1 and *sn*-2 positions of the glycerol backbone. Such modifications of the lipid tails can considerably impact type I NKT cell activation (92). Surprisingly, C16:0/18:1 PG isolated from murine skin, bovine DPG mainly composed of (C18:2/C:18:2)_2_ species and their respective synthetic version have high potency to activate the 14S.10 type II NKT cell hybridoma in a CD1d-dependent manner (58), suggesting that both self- and microbial phospholipids can be recognized by these T cells.

Lysophospholipids are produced after phospholipid hydrolysis by a phospholipase and act as lipid messengers in many physiological processes. These lipids are found in high concentration at inflammatory sites, suggesting a role in the etiology of disorders such as autoimmune diseases, obesity, atherosclerosis, and cancer. Markedly up-regulated in myeloma patients, LPC species were recognized by human type I NKT cell clones (93) and Vα24^−^/Vβ11^−^ type II NKT cells (Figure 1) (68). Chang et al. also reported that human LPC-reactive type II NKT cells predominantly secreted IL-13 in response to LPC, suggesting an immunosuppressive function of these cells (68). LPC isoforms C18:0 and C16:0 were the most potent to activate sulfatide-reactive type II NKT cells compared to the C24:0 isoform (94). Notably, LPC is not recognized by murine type II NKT cells. Similar to LPC, lysosphingomyelin (LSM), which displays the same head group, choline, activated both type I and XV19 type II NKT cells (93, 94). Recently, Zeissig and co-workers showed that lysophosphatidylethanolamine (LPE), which accumulated during hepatitis B virus (HBV) infection, activated type II NKT cells from Jα18-deficient IL-4 reporter mice but not type I NKT cells (53). This activation could be induced by different LPE isoforms, including C16:0, C18:0, and C18:1. Thus, activation of CD1d-restricted T cells in inflammatory contexts where exogenous microbial lipid antigens are not available can result from recognition of inducible self-lipids.

Thus, several self-lipids are recognized by subsets of type II NKT cells and recognition of these lipids is important for type II NKT cell function. Indeed, during microbial infection and non-infectious inflammatory conditions, up-regulation and presentation of self-lipids or stimulatory lyso forms converts innate danger signals into activation of type II NKT cells.

#### Microbial and Other Exogenous Ligands

Until recently, no microbial antigen had been identified that could be directly recognized by non-iNKT cells. Based on the ability of human CD1b molecules to present lipid antigens from *Mycobacterium tuberculosis* (*Mtb*) to diverse CD1b-restricted T cells (7) and due to the similarities between CD1b and CD1d intracellular trafficking and localization in humans (95), we investigated *Mtb* lipids for antigens that may be recognized by type II NKT cells. Purification, isolation and structural analysis of *Mtb* polar lipids revealed PG species with different acyl chain combinations (including C19:0/C16:0, C19:0/C16:1, C18:1/C16:0, and C18:1/C16:1) to stimulate several type II NKT cell hybridomas (14S.6, 14S.10, TBA7, VII68, and XV19) in a CD1d-dependent manner (Figure 1) (93). Moreover, PG, diphosphatidylglycerol (DPG or cardiolipin) and PI from *Corynebacterium glutamicum* (*Cg*), with C18:1/C16:0, (C18:1/C16:1)_2_, and C18:1/C16:0 fatty acid composition, respectively, also activated type II NKT cells, in particular 14S.6, 14S.10, and TBA7 hybridoma cells. In addition, *Mtb* and *Cg* apolar lipids activated type II NKT cell clones in a CD1d-dependent manner, but specific stimulatory lipid species have not yet been identified from this lipid fraction. Similarly, Wolf and colleagues recently demonstrated that PG and DPG isolated from *Listeria monocytogenes* (*Lm*) were also potent ligands for type II NKT clones (TBA7 and 14S.10 especially) (59). Interestingly, whereas in both studies neither PG nor DPG from *Mtb*, *Cg*, and *Lm* succeeded in activating type I NKT cell hybridomas, DPG can be recognized by a subset of CD1d-restricted γδ T cells. Indeed, hepatic and splenic γδ T cells secreted IFN-γ and RANTES (CCL5) in response to DPG in a CD1d-dependent manner (74).

Phospholipids are also found in plant, especially in pollen, which are an important source of environmental allergens. Extracted from cypress grains, phospholipids, as PC in particular, stimulated non-Vα24 NKT and γδ T cells from cypress pollen-sensitive subjects in a CD1d-restricted fashion (72, 73, 96). However, the structure of the acyl chain combination of the stimulatory PC from pollen has not been defined. Interestingly, non-lipidic small molecules can also be associated with CD1d proteins with low affinity. These sulfur-containing molecules, including phenyl 2,2,4,6,7-pentamethyldihydrobenzofuran-5-sulfonate (PPBF) and structurally related compounds (Figure 1), activated human non-Vα24^−^ CD1d-restricted T cells that expressed a Vα2/Vβ21 TCR (97). Thus, in addition to the prominent recognition of self-lipid antigens, non-invariant CD1d-restricted T cells also recognize a range of microbial and exogenous lipids presented by CD1d.

### CD1d/antigen recognition

Considering the differences in TCR usage and antigen specificities among the different CD1d-restricted T cell populations, unraveling the molecular mechanisms by which their TCRs recognize CD1d/antigen complexes is central to understanding their functions. Indeed, recent structural analyses have brought to light key differences in antigen/CD1d recognition by the TCRs of type I NKT cells, Vα24^−^/Vα10^+^ NKT cells, type II NKT cells, and γδ T cells.

**Figure 3:**
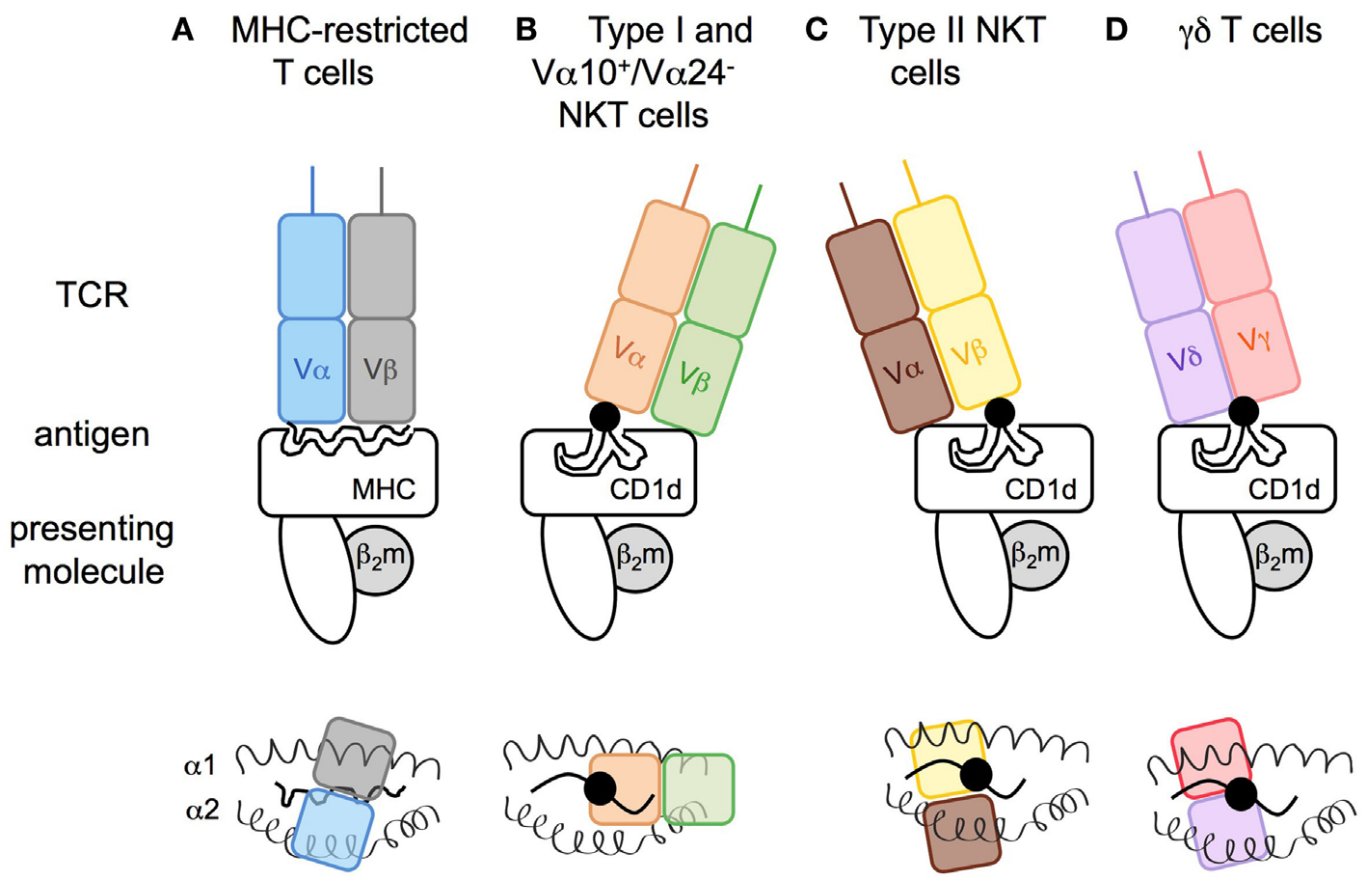
**Modes of antigen recognition for CD1d-restricted T cell receptors**. The mode of TCR docking on **(A)** MHC I or **(B–D)** CD1d antigen complexes. **(A)** Whereas both TCR α- and β-chain of conventional T cells are in contact with the peptide (snaky black line) and the major histocompatibility complex class-I (MHC I), **(B)** the type I NKT cell TCR α-chain interacts with the lipid head group of the glycolipid (black circle) that is anchored in the CD1d molecule by its acyl chains (black lines), and the β-chain is in contact with the CD1d molecule leading to a marked parallel binding position. In an opposite manner, the **(C)** TCR of type II NKT or **(D)** γδ T cells dock in a perpendicular position resulting of the β- or γδ-chains interacting with the polar head group of the antigenic lipid. Upper panels: view from the front. Lower panels: view from above the TCR.

The invariant type I NKT cell TCR docks over the F′ pocket of CD1d in an orthogonal conformation (Figures 3A,B) (98, 99). Only the α-chain, via the complementary-determining region 1α (CDR1α) and CDR3α, is in contact with the head group of α-linked glycolipids such as α-GalCer, and thus has an important role in specificity for, and recognition of, glycolipids. CDR3β and CDR2β loops stabilize the complex by interacting with the CD1d molecule. Variations in TCR β-chain usage cause subtle structural modifications in the conformation of the TCR α-chain, which indirectly contribute to the preferential recognition of some antigens or modulate the affinity for antigen/CD1d complexes (99–101). For the recognition of β-linked ligands, type I NKT TCRs operate through induced-fit molecular mimicry by pushing the β-linked headgroup into a flattened position that is similar to that of the headgroup of stimulatory α-linked lipids (102–104). Therefore, the mode of antigen/CD1d recognition by type I NKT TCRs has been compared to the function of pattern-recognition receptors that interact in an innate-like and conserved manner with ligands that represent microbe-associated molecular patterns or endogenous stress-induced ligands (105, 106).

The Vα24^−^ and Vα10^+^ NKT TCRs from humans and mice, respectively, display very similar conformation compared with type I NKT TCRs in complex with α-GalCer/CD1d, except that the Vα10^+^ CDR2α loop is also in contact with the antigen (46) and that the Vα24^−^ CDR1α loop interacts with the galactose headgroup highly contributing to the TCR fine specificity for the antigen (44).

In contrast, structural analysis of the tri-molecular sulfatide/CD1d/XV19 TCR complex showed that this type II NKT TCR docks above the extreme end of the A′ pocket and adopts a parallel mode vis-a-vis of the CD1d molecule, similar to the interaction of conventional T cell with MHC molecules (Figures 3A,C). Both TCR α- and β-chains interact with CD1d and CDR1β and CDR3β loops confer recognition and discrimination for the antigen (107, 108). Unlike the recognition of β-linked glycolipids by the type I NKT TCR, the type II NKT TCR does not flatten the sulfatide headgroup during ligation. Thus, differences at the TCR-CD1d/antigen interfaces between type I and type II NKT cells provide insights into the molecular basis for the different ligand specificities and highlight how altered TCR use results in a differing docking solution on a monomorphic antigen-presenting molecule. The extent to which the TCR diversity of type II NKT cells results in different docking solutions for distinct TCRs remains to be determined.

Similar to type II NKT TCRs, the TCRs of CD1d-restricted γδ T cells dock above the A′ pocket and all CDRδ loops (CDR1δ, CDR2δ, CDR3δ) are in contact with CD1d (Figure 3D). Notably, the CDR3δ loop also contacts the antigen. Whereas sulfatide recognition requires a central position of the δ-chain above the lipid portal with no contribution of the TCR γ-chain (77), the interaction with α-GalCer involves γ-chain/CD1d contacts and a docking mode closer to the extreme end of the A′ pocket (75). Thus, γδ T cells can utilize a variety of interactions with their TCR γ- and δ-chains to recognize lipid/CD1d complexes.

### CD1d antigen presentation

**Figure 4:**
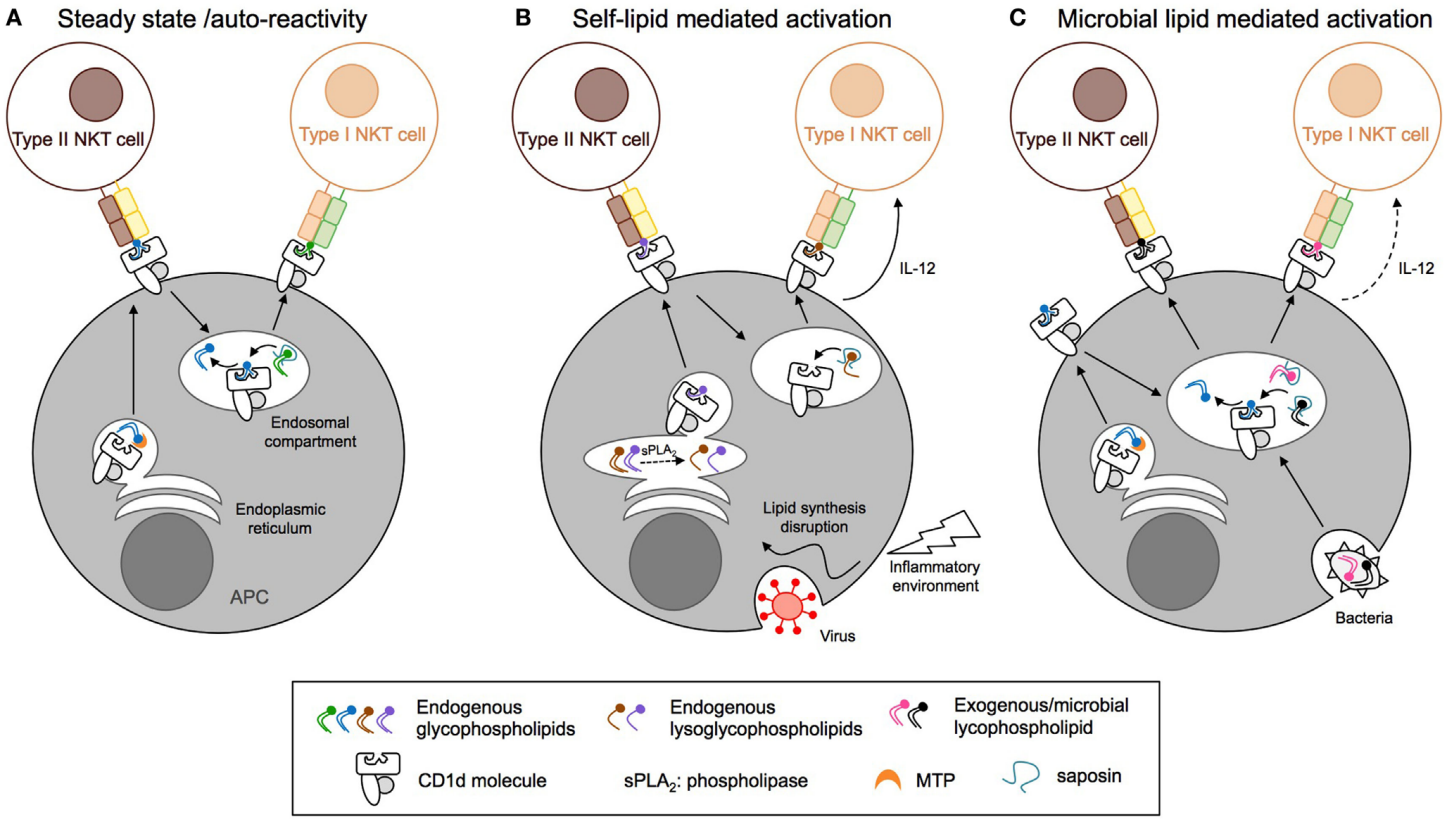
**Distinct antigen presentation pathways regulate the activation of CD1d-restricted T cells**. **(A)** At steady state, endogenous lipids are loaded onto CD1d in the endoplasmic reticulum (ER) compartment with the help of microsomal triglyceride transfer protein (MTP) and directly activate type II NKT cells. Activation of type I NKT cells requires CD1d endocytosis and replacement of the bound lipids by antigens present in the endosomal compartment, a process that is edited by saposins. **(B)** Inflammation and viral infection lead to alterations of the endogenous lipid synthesis and increased expression of secretory phospholipase (sPLA_2_). This results in endogenous phospholipid hydrolysis and generation of stimulatory lysophospholipids or enhanced synthesis and/or decreased degradation of stimulatory lipids for type I and type II NKT cells, respectively. **(C)** Pathogenic bacteria, fungi, or parasites express microbial lipids that can be loaded onto CD1d molecules in endosomal compartments, resulting in the activation of type I and II NKT cells. APC, antigen-presenting cell; NKT, natural killer T cell; MTP, microsomal triglyceride transfer protein.

In humans, the CD1 family is composed of five isoforms divided into two groups: CD1a, CD1b, CD1c, and CD1e forming group 1 and CD1d forming group 2. In mice, only the CD1d isoform is expressed. CD1 molecules are transmembrane glycoproteins that display structural similarities to MHC class-I: one heavy chain composed of three domains (α1, α2, and α3) non-covalently linked to β2-microglobulin via the α3 domain. The CD1d ligand-binding site is composed of α1 and α2 helices, which form two deep hydrophobic pockets, called A′ and F′. The pockets accommodate hydrocarbon chains of glycolipid antigens leading to the protrusion of the lipid head group on the surface and thus its accessibility to the TCR of CD1d-restricted T cells (109, 110). The α3 domain connects the ligand-binding region to a transmembrane domain, followed by a short cytoplasmic tail. The cytoplasmic tail of human and mouse CD1d is critical for its intracellular localization as deletion of a tyrosine-based motif encoded in it interrupts CD1d recycling between the plasma membrane and endolysosomal compartments (Figure 4) (111, 112). Moreover, the internalization of murine CD1d and its subsequent lysosomal localization require the binding of the adaptor protein complex AP-3 to the tyrosine-based motif (113, 114). Interestingly, human CD1d fails to bind the AP-3 complex, unlike human CD1b (113). In humans, CD1d trafficking from ER to cell surface and lysosomes is orchestrated by its physical interaction with MHC II and invariant chain (115, 116). By extension from its role in CD1d trafficking, the tyrosine-based motif in the cytoplasmic tail of CD1d is also critical for type I NKT cell function. Indeed, its deletion impaired type I NKT cell development and activation by endogenous and exogenous antigens such as α-GalCer (60, 112, 117). In contrast, CD1d with a truncated cytoplasmic tail is still capable of activating type II NKT cells and does not impair their development, suggesting that loading of type II NKT cell self-antigens onto CD1d occurs within the ER (60, 111, 112). Recently, Shin and co-workers have shown that three consecutive arginine residues between the transmembrane region and the cytoplasmic tail are involved in the intracellular trafficking of CD1d and the presentation of endogenous glycolipids to both type I and type II NKT cells (118). Selection and editing of CD1d-bound lipids is also influenced by accessory lipid-binding and -loading molecules such as the microsomal triglyceride transfer protein (MTP) and saposins that facilitate the formation of lipid/CD1d complexes in the ER and throughout the endocytic pathway, respectively (Figure 4). The ability of CD1d molecules to differentially stimulate type I versus type II NKT cells based on their distinct intracellular trafficking routes is associated with loading of distinct self-lipids onto CD1d. Indeed, using lipid elution and mass spectrometry, Yuan et al. demonstrated that ER-retained CD1d molecules associated with phosphatidylcholine (PC), whereas CD1d molecules that trafficked through the secretory pathway were loaded with sphingomyelin for which terminal synthesis occurs within the Golgi apparatus (119). Interestingly, recycling CD1d molecules carried a combination of both sphingomyelin and PC but also lysophospholipids that result from lipid degradation by phospholipase in lysosomes. In contrast to the presentation of endogenous lipid antigens, activation of type II NKT cells by exogenous self- or microbial lipids requires cellular lipid uptake and intersection of lipid loading and intracellular CD1d trafficking in secretory and/or lysosomal compartments (93, 120). Thus, different intracellular antigen presentation pathways result in formation of stimulatory lipid/CD1d complexes for type I and type II NKT cells.

### Cytokine- and NK receptor-mediated activation

Activation of naïve MHC-restricted T cells is controlled by TCR signals that result from highly specific recognition of peptide antigen/MHC complexes in combination with receptor-mediated co-stimulatory signals, whereas the immediate cytokine milieu is critical to guide the differentiation of T cell effector functions. In contrast, innate T cells such as type I NKT cells have adopted a strategy for their activation that integrates TCR-mediated signaling and stimulation by pro-inflammatory cytokines to result in rapid activation (16). Dependent on the context and on the affinity of the CD1d-presented lipid antigens, one signal prevails over the other. For example, a high-affinity lipid antigen such as a potent induced self-lipid or microbial lipid can result in strong and predominantly TCR-mediated activation with no or only limited need for cytokine stimulation. In contrast, presentation of low-affinity microbial or self-antigens requires stimulation with antigen-presenting cell-derived inflammatory cytokines to result in overt NKT cell activation. This cytokine-mediated stimulation can be so strong that it alone results in overt activation, obviating the need for TCR-mediated stimulation altogether. This mechanism ensures activation of type I NKT cells early in an ensuing inflammatory response during infection even when no microbial CD1d-presented lipids are expressed, as is the case, for example, during viral infections, and similar mechanisms may explain type I NKT cell activation in tumor immunity and autoimmune diseases (121, 122). At steady state, type I NKT cells highly express a wide range of cytokine receptors and have therefore the ability to respond to multiple cytokines like IL-12 (123, 124), IL-18 (125), and IL-23 alone or in combination with IL-1β (126, 127), IL-25 (30), and IFN type 1 (86). Depending on the cytokines present in the inflammatory environment, type I NKT cells secrete different cytokines such as IFN-γ in response to IL-12, IL-18 or IFN type 1, IL-17 with IL-23, and IL-22 with IL-23/IL-1β in combination.

Rolf and co-workers have compared by gene expression profiling the expression of cytokine receptors between type I NKT cells and type II NKT cells from 24αβ TCR transgenic mice (128). Interestingly, whereas *IL-18r1*, *IL-18rap* (IL-18 receptor associated protein), and *IL2ra* (CD122) were expressed at high and similar levels in both subsets, type I NKT cells present a higher expression of *IL12rb1* (up to 3-fold) and *IL2ra* (CD25) (up to 11-fold). Compared to conventional naïve CD4^+^ T cells, both type I and type II NKT cells displayed low levels of *IL1r2*, *IL6ra*, and *Ifngr2*. Recently, IL-25 has been shown to activate type II NKT cells *in vivo* and promote their production of IL-13 through IL17RB (129, 130). This suggests that, similar to type I NKT cells, type II NKT cells can respond to stimulation with inflammatory cytokines. Interestingly, during HBV infection activation of type II NKT cells was primarily driven by recognition of CD1d-presented lysophospholipids and largely independent of IL-12 signaling, whereas activation of type I NKT cells was significantly reduced in the absence of IL-12 (53). Whether indirect mechanisms also contribute to the activation of CD1d-restricted γδ T cells remains to be determined.

Initially named owing to their expression of NK1.1, NKT cells express a multitude of both activating (for example, NKG2D, NK1.1, CD160, NKR-P1A, NKp46) and inhibitory (Ly49c, Ly49G2, NKG2A, 2B4) NK receptors that play an important role in their activation and regulation. For example, stimulation of NK1.1 and NKG2D was sufficient to directly activate type I NKT cells (131, 132), and NKG2D–ligand interactions were critical for type II NKT cell-mediated disease induction in a mouse model of HBV infection (133). At steady state, both NKT cell subsets highly express *Klrk1* (NKG2D), *Klrb1c* (NK1.1), and *CD160* but only type II NKT cells display high levels of *Klra3* (Ly49c), *Klra7* (Ly49G2), and in a lesser manner *Ncr1* (NKp46). For their part, type I NKT cells express higher levels of *Klrb1a* (NKR-P1A) and *Klrc1* (NKG2A) (128).

Thus, in order to overcome the limitations of restricted antigen specificity that result from limited TCR diversity and the recognition of a monomorphic antigen-presenting molecule, both type I and type II NKT cells appear able to integrate stimulatory signals provided by TCR-mediated stimulation and inflammatory cytokines with modulation by NK receptor-signaling, to ensure their rapid activation in various infectious and inflammatory contexts.

## Functions of Type II NKT Cells

In contrast to the large body of literature previously mentioned that has documented a role for type I NKT cells in various pathological states, progress in understanding the role of other CD1d-restricted T cell populations has been hampered by the limited ability to track these cells and the lack of models to assess their function *in vivo*, in particular for Vα24^−^/Vα14^−^ NKT cells and CD1d-restricted γδ or δ/αβ T cells. However, several lines of evidence suggest that type II NKT cells can contribute to and modulate a range of immune responses, occasionally in opposing roles to type I NKT cells.

### Microbial infections

#### Viral Infections

**Figure 5:**
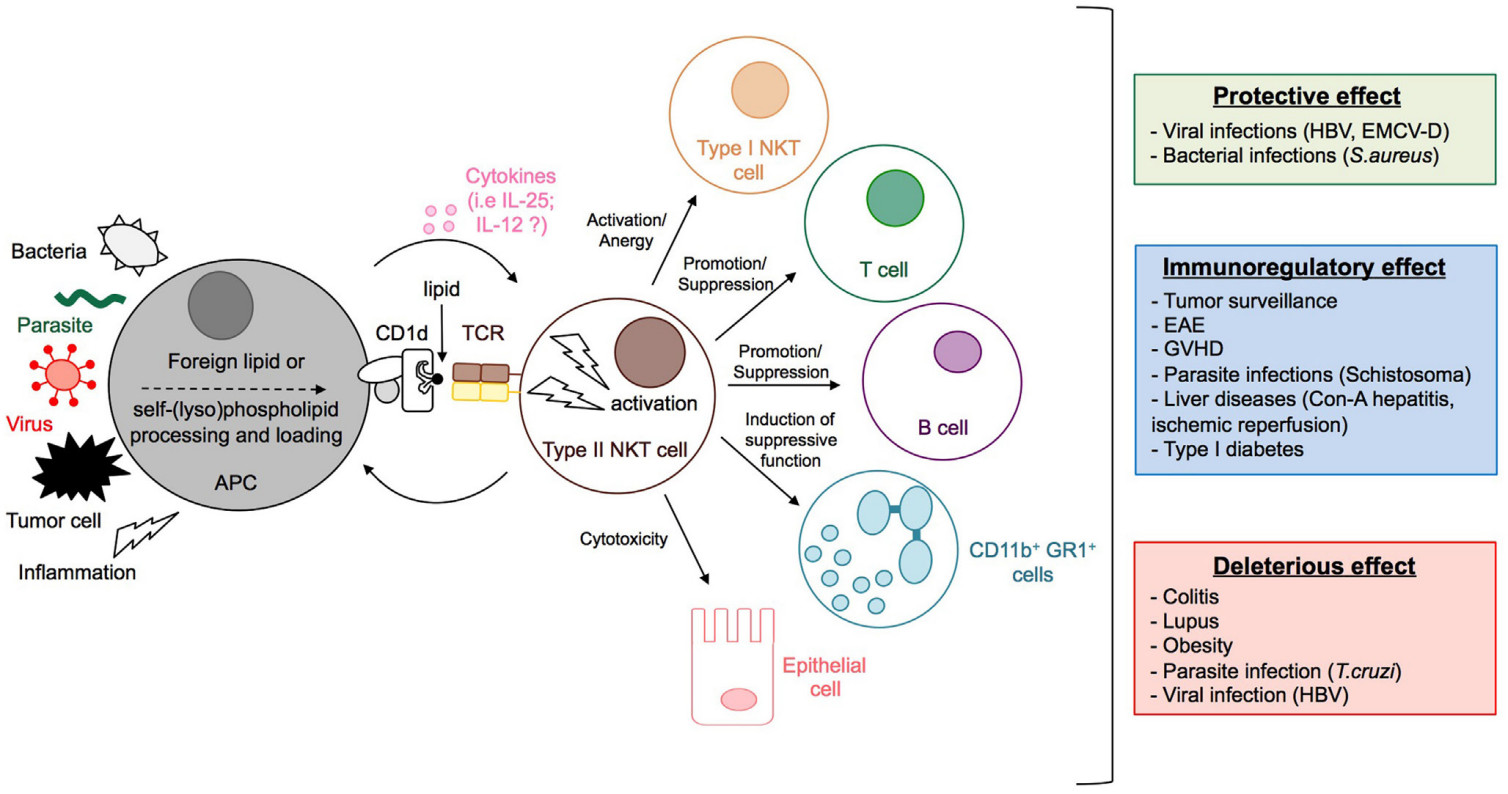
**Activation and functions of type II NKT cells**. The stimulation by microbial lipids (infection with bacteria or parasites) or self-lipid antigens (viral infection, inflammation, or tumor cell) in combination, or not, with cytokines, leads to the activation of type II NKT cells. Dependent on the context, type II NKT cells promote or suppress immune responses by modulating the activation of type I NKT and T cells, granulocytes, and B cell antibody responses, and can even impact directly the integrity of tissues, for example, by killing epithelial cells. Whereas type II NKT cells can contribute to protective immunity during viral and bacterial infection, they can have immunomodulatory effects that reduce disease pathology during experimental autoimmune encephalomyelitis (EAE), GVHD, inflammatory liver disease, type 1 diabetes, or parasitic infection. In contrast, type II NKT cells can also have deleterious effects during tumor surveillance, colitis, lupus, obesity, and parasitic infection.

The first indication that type II NKT cells can contribute to protective immunity during viral infection came from studies in mice using diabetogenic encephalomyocarditis virus-induced pathology (EMCV-D) that is characterized by hind-limb paralysis and impaired glucose-tolerance resulting from virus cytopatic effects on neuronal cells and islet cells, respectively. Whereas EMCV-D infection resulted in similar disease severity in WT and Jα18^−/−^ mice, CD1d^−/−^ mice exhibited a higher incidence and exacerbated severity of the disease, suggesting a protective role of type II NKT cells during this infection (Figure 5) (134). However, this protective function was not unique to type II NKT cells since activation of type I NKT cells by α-GalCer provided protection of WT mice during EMCV-D infection. An additional example of the protective potential of type II NKT cells during viral infection comes from studies in humanized SCID mice that were infected with HIV-1, in which administration of sulfatide resulted in reduced viral replication (135). Thus, natural or sulfatide-induced activation of type II NKT cells during viral infections can enhance protective innate and adaptive immune responses (Figure 5).

Infection with human HBV is a common cause of acute and chronic liver injury, including cirrhosis and hepatocellular carcinoma. The immune response during HBV infection plays a dual role, by eliciting tissue damage in response to viral antigens and by controlling viral replication. Mice engineered to express HBV envelope proteins in hepatocytes, or a terminally redundant HBV DNA construct that results in intrahepatic HBV replication has been used to study HBV infection in mice, and showed that type II NKT cell responses contributed to acute hepatitis and tissue injury in a CD1d- and NKG2D-dependent manner (133, 136). More recently, Zeissig and colleagues used HBV-expressing adenoviral particles to reproduce HBV infection in mice and uncovered a protective role for type II NKT cells (53). Type II NKT cell activation in response to HBV was dependent on the expression of CD1d and MTP (53) which transfers endogenous phospholipids onto CD1d (137, 138), suggesting an essential role for endogenous lipid presentation. Furthermore, as observed both in patients with viral hepatitis and HBV-infected mice, HBV infection increased the transcription of the secretory phospholipase A_2_ (sPLA_2_) (139) and expression of one of its substrates, PE. Interestingly, the analysis of microsomal lipids revealed an increase of LPE isoforms able to activate type II NKT cells. Type II NKT cell activation during HBV infection was predominantly based on LPE recognition, as IL-12 neutralization had little effect. By contrast, type I NKT cells did not recognize LPE but required IL-12 and MTP, suggesting that their activation occurred after type II NKT activation and APC maturation. In addition to their critical role in initiating an innate immune response against HBV in this model, type II NKT cells appear to also be involved in modulating the generation of protective adaptive immune responses. Indeed, HBV-infected CD1d^−/−^ mice (lacking both type I and type II NKT cells) displayed significantly reduced activation of CD4 and CD8 T cells, whereas Jα18^−/−^ mice (lacking only type I NKT cells) showed only reduced activation of CD4 T cells. This type II NKT cell-dependent reduction of adaptive CD8^+^ T cell responses during HBV infection led to a defect in viral control and resulted in chronic inflammation (53). Thus, in mouse models of HBV infection, type II NKT cells can both contribute to immune-mediated tissue damage and to the innate and adaptive immune responses that control viral replication (Figure 5). In humans, type II NKT cells that produced large amounts of IFN-γ, but little IL-13 or IL-4, accumulated in the liver during hepatitis C (HCV) infection, but their role during infection remains to be determined (140, 141).

#### Bacterial Infections

As described above, type II NKT cells are activated in response to diverse bacterial antigens, suggesting a role for these cells in host defense against these bacterial pathogens. During sepsis, the immune response consists of a primary phase characterized by a potentially lethal cytokine burst induced by activated leukocytes, and a secondary latent phase where host defense is reduced. Despite the rapid activation of type I NKT cells (increased CD69 expression, proliferation) after *Staphylococcus aureus* inoculation, Jα18^−/−^, CD1d^−/−^, and WT mice presented comparable rates of mortality, suggesting that neither type I nor type II NKT cells play a significant protective or deleterious role in *S. aureus* sepsis (142). However, administration of sulfatide concomitant with *S. aureus* inoculation improved the survival rate of mice (Figure 5). Interestingly, the protective effect of sulfatide was CD1d-dependent and type I NKT cell-independent, suggesting that activation of sulfatide-reactive type II NKT cells is essential and sufficient for this protective effect. Sulfatide treatment was accompanied by a decrease of the inflammatory cytokines TNF-α and IL-6. Thus, activated sulfatide-reactive type II NKT cells reduced the cytokine storm of the primary hyper-reactive phase during *S. aureus* sepsis while maintaining an adequate immune response to limit bacterial growth and clearance.

During *Mtb* infection, type I NKT cells produced IFN-γ through stimulation with IL-12/IL-18 and inhibited intracellular bacterial replication by their CD1d-restricted secretion of GM-CSF (143, 144). As described above, several type II NKT hybridomas were activated by *Mtb*-infected APC through the specific recognition of *Mtb* phospholipids (93), suggesting that, similar to type I NKT cells, type II NKT cells may also contribute to protective immunity during *Mtb* infection. Similarly, given the recognition of *Lm* antigens by type II NKT cells, the increased bacterial burden in CD1d^−/−^ during *Lm* infection as well as the ameliorated *Lm* infection following treatment with blocking anti-CD1d antibodies suggests that type II NKT cells may contribute to protective immunity or have a regulatory function during *Lm* infection (145, 146).

#### Parasitic Infections

Schistosomiasis is a chronic parasitic disease caused by the extracellular parasite *Schistosoma*, and a strong Th2 response, triggered by parasite eggs, is essential for host survival. CD1d-deficient mice developed a markedly reduced Th2 response during schistosomiasis (147) indicating an important role of CD1d-restricted T cells in the development of a protective immune response. Whereas type I and type II NKT cells were not essential during the very early phase (1 and 4 weeks) of infection, the two subsets played distinct but complementary roles during the acute phase (7 and 12 weeks) of infection (148). Indeed, type II NKT cells promoted the Th2 response as their lack induced a decrease of Th2 cytokines (IL-4, IL-5, and IL-10) and specific IgG1 production (Figure 5), a feature also described in alum-induced humoral immune response (149). On the other hand, secretion of IFN-γ by type I NKT cells contributed to the production of specific IgG2b, a marker of Th1 responses. Interestingly, schistosome egg-sensitized DC activated type I but not type II NKT cells and this activation required the presentation of self-antigen rather than parasite-derived antigens or TLR2/TLR3 engagement (148). Nonetheless, Magalhaes and co-workers have shown that administration of schistosoma-derived LPC or cercaria induced secretion of IL-5 and IL-13 (150) dependent on TLR2, and lead to the recruitment of eosinophils at the site of infection. The cellular source(s) of Th2 cytokines have not been investigated in this model, but considering that LPC is a potent type II NKT cell antigen, it is likely that these cells are activated during infection.

Natural killer T cell subsets also displayed opposing roles during infection with *Trypanosoma cruzi* (151). *T. cruzi* infection causes a chronic inflammatory disease in which the anti-*T. cruzi* immune response that controls the persistent parasites can also contribute to the inflammatory tissue damage of the myocardium and gastro-intestinal tract that causes Chagas disease. Indeed, Jα18^−/−^ mice were more sensitive to infection with *T. cruzi* compared to WT or CD1d^−/−^ mice as indicated by greater morbidity and mortality. The increased susceptibility to infection in the absence of type I NKT cells was accompanied by an enhanced inflammatory response with infiltrates of activated cells (NK and T cells), B cells and DC in lymphoid organs and greater muscle inflammation and pro-inflammatory cytokine secretion (IFN-γ, TNF-α, nitric oxide). Moreover, the humoral response was impaired as shown by a decreased anti-*T. cruzi* IgG2a antibody titer, compared to WT and CD1d^−/−^ mice. This suggests that during *T. cruzi* infection, type II NKT cells augment the inflammatory anti-parasite response, whereas type I NKT cells limit this response (Figure 5).

Together, these examples show that in a range of viral, bacterial, and parasitic infections type II NKT cells can either promote protective innate and adaptive cell-mediated immune responses, or contribute to infection-induced pathology. Understanding the mechanisms that determine these opposing functions of type II NKT cells during infection will be important areas of future research.

### Anti-tumor immunity

Type I NKT cells critically contribute to natural anti-tumor responses, as demonstrated by the prompt growth of spontaneous tumors in type I NKT cell-deficient Jα18^−/−^ mice compared to WT mice (152–154). Furthermore, the activation of type I NKT cells by α-GalCer provides potent effects against hematologic malignancies and solid tumors through their IFN-γ-production and the subsequent activation of DC and NK cells (155, 156). By contrast, sulfatide-activated type II NKT cells repress anti-tumor immunity (Figure 5) (157–159) by abrogating type I NKT activation in response to α-GalCer, in terms of cytokine secretion and expansion (160). Moreover, their IL-13 production, in combination with TNF-α, led to up-regulation of TGF-β secretion by myeloid-derived suppressor cells (MDSC), and resulted in decreased cytotoxic T cell activity (161). Interestingly, in contrast to their notable immunoregulatory role in anti-tumor responses, two recent studies have highlighted the ability of type II NKT cells to contribute to anti-tumor immunity in response to CpG in a B16 melanoma model (54), and by their ability to directly kill lymphoma cells *in vitro* (63). Thus, type II NKT cells can suppress anti-tumor immunity, counteracting the anti-tumor activity of type I NKT cells, but can also contribute to defense against tumor growth. Understanding the factors that determine the role of type II NKT cells in tumor immunity will be critical to harness their potential in novel anti-tumor strategies.

### Autoimmunity and tolerance

Type II NKT cells help maintain tolerance to self-antigens and thereby prevent autoimmune disease. On the other hand, they also can mediate tissue damage and play a pathogenic role in autoimmunity.

#### Experimental Autoimmune Encephalomyelitis

An important role for type II NKT cells has been shown in experimental autoimmune encephalomyelitis (EAE), a mouse model for multiple sclerosis. During EAE, sulfatide-mediated activation of type II NKT cells induced tolerogenic DC polarization, abrogated activation of both type I and type II NKT cells, class-II MHC effector myelin protein reactive T cells, and microglial cells and suppressed autoimmunity (Figure 5) (55, 162).

#### Type 1 Diabetes

NOD mice, which spontaneously develop type 1 diabetes, were protected from disease following sulfatide administration that led to type II NKT cell activation and secretion of IL-10 by DC, resulting in inhibition of type I NKT cells and activation and expansion of diabetogenic T cells (Figure 5) (163). In contrast, in another study using a different experimental approach, sulfatide failed to protect NOD mice from diabetes (164). A non-sulfatide-reactive type II NKT cell subset was also able to protect NOD mice from diabetes by regulating diabetogenic T cells through PD-1/PD-L1 and ICOS/ICOSL pathways (66, 165).

#### Systemic Lupus Erythematosus

Systemic lupus erythematosus (SLE) is an autoimmune disease characterized by antinuclear autoantibodies (IgG2a particularly) and multiorgan injury such as proteinuria and immune complex glomerulonephritis, which is dependent on IFN-γ (166, 167). Whereas α-GalCer administration exacerbated the disease by inducing a strong Th1 response, CD1d-blocking slowed down the development of the disease mediated by a decrease of IgG2a production (167). Interestingly, transfer of splenic CD4^+^, CD8^+^, or DN Vα4.4/Vβ9 type II NKT cells, purified from transgenic mice generated with CD1d-reactive T cells initially derived from irradiated mice, promoted the disease by producing large amount of IFN-γ. By contrast, NKT cells derived from bone marrow and displaying a DN phenotype prevented SLE by secreting large amounts of IL-4 (166).

#### Colitis

Type II NKT cells exposed *in vivo* to high levels of CD1d expression could directly contribute to the spontaneous development of colitis in mice (168). Their harmful role was mediated through their IL-13 secretion induced by IL-25 and their cytotoxic activity against epithelial cells (Figure 5) (56, 129, 169).

### Liver diseases, GVHD, and obesity

Halder and co-workers have shown that presentation of sulfatide by hepatic plasmacytoid DC (pDC) to type II NKT cells led to the recruitment of anergic type I NKT cells, in a IL-12- and MIP-2-dependent manner and prevented concanavalin A-induced hepatitis (170). The same group has also shown that type II NKT cells neutralized the pathogenic role of type I NKT cell during hepatic ischemic reperfusion disease (50) and alcoholic liver disease (94). Indeed, activation of type II NKT cells after sulfatide administration suppressed IFN-γ production by hepatic type I NKT cells and inhibited the recruitment of myeloid cells in the liver that have been shown to enhance injury (Figure 5) (171).

In bone-marrow (BM) transfer, both host-residual and donor-derived NKT cells exert protective functions that are particularly well described in graft-versus-host disease (GVHD). Whereas protection by host-residual T cells was provided by the type I NKT subset, type II NKT cells played critical roles in donor-derived protective effects (172–175). Indeed, on the one hand, adoptive transfer of type I NKT cells or the administration of α-GalCer attenuated GVHD in recipient mice due to the vigorous secretion of IL-4 by type I NKT cells and the subsequent Th2 polarization of the immune response (172–175). On the other hand, donor BM type II NKT cells produced IL-4, like type I NKT cells, but also IFN-γ which induced apoptosis of donor CD4^+^ and CD8^+^ T cells in a Fas-dependent manner (174, 176). Moreover, Exley and co-workers have shown that human CD161^+^ CD1d-reactive BM-derived non-invariant T cells specifically suppress mixed-lymphocytes reaction (MLR) and could induce tolerance to allografts (67).

In obesity, activation of type II NKT cells by lipid excess initiated adipose tissue and liver inflammation leading to obesity (177). By contrast, Hams and colleagues found that type II NKT cells activated by IL-25 regulated weight and glucose homeostasis by recruiting to visceral adipose tissue eosinophils and activated macrophages that are found in lean individuals (130).

Thus, in a context-dependent manner, type II NKT cells provide protective or deleterious effects in a number of diverse diseases including microbial infection, anti-tumor immunity, and autoimmunity (Figure 5), suggesting that these cells may constitute promising therapeutic targets for a broad range of diseases.

## Summary and Future Directions

Significant progress has been made in defining the diversity among CD1d-restricted T cells that, in addition to the widely studied type I NKT cells, include α-GalCer-reactive NKT cells that use non-canonical TCRs, type II NKT cells that use a more diverse repertoire of αβ TCRs, as well as T cells that use γδ or δ/αβ TCRs. Critical differences distinguish the members of the CD1d-restricted T cell family beyond their use of different TCRs, including their antigen specificities and modes of antigen/CD1d recognition. A major hurdle in unraveling the biology of the CD1d-restricted T cell subsets that do not recognize α-GalCer is the difficulty to reliably detect and analyze these cells in humans and mice *ex vivo* and *in vivo*. Discovery of new self- and foreign antigens in combination with strategies and tools to directly detect these cells such as antigen-loaded CD1d tetramers, and availability of suitable small animal models will be necessary to characterize the entire repertoire and to determine the degree of diversity versus oligoclonality among all CD1d-restricted T cell subsets. Furthermore, it is currently unknown to what extent the differences in TCR usage and antigen specificity of these subsets correlate with phenotypic attributes or immunologic functions that are distinct from those of type I NKT cells. For example, in anti-tumor immunity and in response to microbial infection, type II NKT cells have been shown to have opposing functions to those of type I NKT cells. In addition, while many non-invariant CD1d-restricted T cells appear to display a type I NKT cell-like activated/memory phenotype, several studies have indicated that CD1d-restricted T cells exist that are more similar to conventional MHC-restricted T cells. Importantly, it remains to be determined if truly naïve CD1d-restricted T cells exist that can mount antigen-specific memory T cell responses following primary stimulation and expansion. Given their ability to recognize bacterial antigens including ones expressed by *Mtb*, this would provide a rationale to target these cells in vaccine strategies. In analogy to the functionally distinct type I NKT cell subsets that comprise NKT1, NKT2, NKT17, and NKT_FH_ cells, the Th2-bias observed among some human type II NKT cells and following sulfatide administration, and the discovery of T_FH_-like type II NKT cells in humans and mice suggest that functionally distinct subsets may also exist among non-invariant CD1d-restricted T cells. Correlation of such functional differences with antigen specificity would allow therapeutic targeting of these cells with activating or inhibitory ligands.

Several examples illustrate that non-invariant NKT cells, in particular type II NKT cells, critically contribute to the immune responses in a wide range of diseases, including microbial infection, anti-tumor immunity, and autoimmunity. Central to our understanding of the varied roles of CD1d-restricted T cells is unraveling the mechanisms that regulate their activation in these diverse conditions. During infection, recognition of inducible self-lipid antigens in combination with stimulation by the inflammatory milieu through cytokines, or direct recognition of microbial antigens results in the rapid activation of CD1d-restricted T cells. How these T cells distinguish between self- and microbial lipids, whether activation by antigens with different affinities results in distinct functional outcomes, and whether recognition of inducible self-antigens extends to non-infectious conditions such as tumor immunity or autoimmunity are currently not known. Defining the context-dependent mechanisms of activation, decrypting the disease-specific balance of protective and potentially harmful functions of the different CD1d-restricted T cell populations and a better understanding of their effector functions and interactions with other immune cells present significant ongoing challenge and will ultimately determine the success of targeting CD1d-restricted T cells for preventive or therapeutic interventions.

## Conflict of Interest Statement

The authors declare that the research was conducted in the absence of any commercial or financial relationships that could be construed as a potential conflict of interest.
